# Reliability and validity of a perinatal depression screening instrument in rural Mali

**DOI:** 10.1016/j.ssmmh.2021.100059

**Published:** 2022-01-02

**Authors:** Molly E. Lasater, Madeleine Beebe, Nicole E. Warren, Peter J. Winch, Fatoumata Soucko, Mariam Keita, Seydou Doumbia, Sarah M. Murray

**Affiliations:** a Social and Behavioral Interventions Program, Department of International Health, Johns Hopkins Bloomberg School of Public Health, 615 N Wolfe St, Baltimore, MD, 21205, USA; b School of Nursing, Johns Hopkins University, 525 N Wolfe St, Baltimore, MD, 21205, USA; c Department of Public Health, Faculty of Medicine and Odontostomatology, University of Sciences, Techniques and Technology of Bamako, Mali; d Department of Mental Health, Johns Hopkins Bloomberg School of Public Health, 624 N Broadway, Baltimore, MD, 21205, USA

**Keywords:** Perinatal depression, Mental health, Maternal health, Sub-saharan Africa, Validity, Psychometrics

## Abstract

**Background:**

In order to reduce the burden of perinatal depression in low- and middle-income countries, health systems must be able to identify and treat women suffering from depression. The objective of our study was to develop a locally valid and reliable screening instrument for use in identifying pregnant women and mothers of young children with a local depression syndrome, *dusukasi*, in rural Mali.

**Methods:**

We administered a locally adapted screening instrument containing items from the Edinburgh Postpartum Depression Scale (EPDS) and Hopkins Symptom Checklist (HSCL-25) to 180 pregnant women and mothers of children under age 2 in Sélingué, Mali to assess the instrument’s psychometric properties and validity. Item Response Theory was used to develop an abbreviated version of the measure and the validity and psychometric properties of this shortened version were compared with the full-length scale.

**Results:**

The full 28-item scale exhibited a single factor structure with good internal consistency (Cronbach’s alpha = 0.92). Women who self-identified as suffering from *dusukasi* (n = 87) in a known groups analysis to assess construct validity had significantly higher depression and anxiety symptom scores (p < 0.0001) and functional impairment scores (p < 0.0001) compared to women not reporting *dusukasi* (n = 93). The shortened 16-item scale performed as well as the full scale in identifying women with *dusukasi*.

**Conclusions:**

Construct validity of our adapted screening instrument was supported for identifying *dusukasi* in rural Malian women. Our methodology can be applied in other settings to develop similarly valid screening instruments for perinatal depression.

## 1. Introduction

Common perinatal mental disorders (CPMDs), i.e. depression and anxiety, are estimated to be experienced by 15.6% of pregnant women and 19.8% of women who have recently given birth in low- and middle-income countries (LMIC) (Fisher et al., 2012). Risk factors for CPMDs in LMIC include low educational attainment, poverty, mood changes during pregnancy, intimate partner violence, unemployment, and poor newborn health (Abiodun, 2006; Adewuya, 2006; Brown et al., 2020; Ebeigbe & Akhigbe, 2008; Fekadu Dadi et al., 2020; Garman et al., 2019; Gausman et al., 2020; Patel, 2007; Rahman et al., 2013; van Heyningen et al., 2019; Weobong et al., 2015). Beyond causing suffering and impairing women’s functioning, negative health and developmental outcomes have been observed among children of women with CPMDs (Bass et al., 2008; Fekadu Dadi et al., 2020; Gureje et al., 2015; Santoro & Peabody, 2010; Shidhaye & Giri, 2014) including malnutrition, stunting, elevated rates of illness, and cognitive and emotional delays (Mughal et al., 2019; Surkan et al., 2011; Walker et al., 2007).

The perinatal period represents an opportunity for healthcare providers to identify women and infants at risk of poor health outcomes due to CPMDs (Lomonaco-Haycraft et al., 2019; Patel et al., 2009). As such, screening tools that can accurately identify perinatal women in need of mental health services are vital to addressing the burden of CPMDs, particularly in LMIC. However, standard screening instruments for depression, such as the Beck Depression Inventory or Hopkins Symptom Checklist, lack criterion and face validity for postpartum depression (Cox, 2019). The Edinburgh Postnatal Depression Scale (EPDS) (Cox et al., 1987) was developed to address this gap and is now one of the most widely used screening instruments for CPMDs globally (Cox, 2019).

Although CPMDs are a global phenomenon, clinical presentation can vary widely (Bass et al., 2007; Haroz et al., 2016a; Kirmayer, 2001; Rodrigues et al., 2003); thus, diagnostic criteria cannot be presumed to be equally applicable across cultural settings (American Psychiatric Association, 2013; Bass et al., 2007). Moreover, functional impairment, a key criterion for diagnosing depression, necessitates locally tailored measures of functioning related to daily living tasks which may vary substantially cross culturally. Yet, of 25 studies identified in a review of perinatal depression instrument validation studies in sub-Saharan Africa, only three instruments were developed for use within a specific cultural setting (Bass et al., 2008; Kaaya et al., 2008; Nhiwatiwa et al., 1998; Tsai et al., 2013). Further, limited studies have been conducted to assess evidence of validity of existing instruments in LMIC; a systematic review identified only 9 antenatal and 27 postnatal validation studies of mental health instruments in LMIC (Ali et al., 2016).

When using instruments developed in high-income countries (HIC) to assess CPMDs in LMIC, the quality of instrument translation and adaption process is of concern (Haroz et al., 2016b). Most adaptations of instruments developed in high income countries for assessment of CPMDs in LMIC have predominantly consisted of translation of tools through wording modifications (Abrahams et al., 2019; Baggaley et al., 2007; Chibanda et al., 2010; Hanlon et al., 2008; Joshi et al., 2020; Velloza et al., 2020), but items were not added or removed based on local relevance. For example, while the EPDS has been translated into over 60 languages and its validity assessed in 12 studies spanning world regions, only one study of its validity included pre-testing for comprehensibility in the translation process (Shrestha et al., 2016). Therefore, it is not surprising that translated versions of the EPDS have demonstrated lower discriminant validity for correctly identifying CPMDs than the original English version (Gibson et al., 2009; Kozinszky & Dudas, 2015; Shrestha et al., 2016). Cox, one of the original authors of the EPDS, cautions that at times the use of the EPDS in research, and community and perinatal services can be suboptimal, and occasionally dangerously misleading, ultimately highlighting the potential need for CPMD screening alternatives to the EPDS, particularly within LMIC (Cox, 2017).

In Mali, fertility and both infant and child mortality are high (CPS/SSDSPF, 2014), and risk factors for CPMDs, including female genital cutting, intimate partner violence, and polygamy are prevalent (CPS/SSDSPF, 2014; Bove et al., 2014; Slegh et al., 2013). Previous qualitative work in rural Mali uncovered a local syndrome, *dusukasi* (“crying heart”) among perinatal women (Lasater et al., 2018), and some signs and symptoms of which map onto diagnostic criteria for major depressive disorder (American Psychiatric Association, 2013), while others did not (e.g. “feeling your heart is broken or pained”) (Lasater et al., 2018)., To date no CMPD screening instruments have been adapted or validated for use in Mali. In this paper, we present the process for locally adapting and assessing the construct validity of existing instruments to identify and measure *dusukasi*, a local perinatal depression syndrome in rural Mali. While in studies of depression, the perinatal period typically ranges from pregnancy to one year post-delivery (Leung & Kaplan, 2009), we included pregnant women and mothers of children under age two to reflect the likelihood that most women have one or more children under two. Given that the feasibility of administering perinatal depression screeners is of central importance in LMICs, where screening efforts are constrained by human resources shortages, we also sought to create a brief instrument to improve access to mental health care during the perinatal period in Mali where mental health services are very limited (Patel et al., 2009; CPS/SSDSPF, 2014; Rochat et al., 2013). Recent literature has suggested that commonly used scales of symptoms of mental disorders can be shortened without the loss of highly relevant items or measurement precision (Chiesi et al., 2018; Haroz et al., 2020; Xia et al., 2019). Such instruments are critically important as health care providers in Mali and similarly low resource settings work to operationalize the global imperative to screen for and prevent CPMDs.

## 2. Methods

The present study was embedded within a larger mixed-methods investigation of the cultural-linguistic landscape of perinatal mental health constructs in Sélingué, Mali (Lasater et al., 2018). This study was approved by the Institutional Review Board at the Johns Hopkins Bloomberg School of Public Health, and the University of Sciences, Techniques & Technologies of Bamako.

### 2.1. Study site

Fieldwork was completed in September and October 2016 in Sélingué, an impoverished southwestern region, approximately 140 miles from the capital, Bamako. Women in Sélingué are largely illiterate and receive minimal, if any, formal education. During data collection, Sélingué health district had one referral hospital and ten community health centers.

### 2.2. Respondents and procedures

We worked with two community health centers, Binko and Tieguecourouni, to compile a list of 13 villages and 17 community health workers (CHWs). CHWs were asked to recruit 12–14 perinatal women living in their same community, whom they knew well, over age 18, and either (a) were pregnant or (b) had given birth within the past two years. We also asked CHWs to select women who faced “serious problems, for example a difficult family situation”, and others who had “fewer difficulties”, to yield a sample with an approximately even split between cases and non-cases of *dusukasi*. The women identified by CHWs were different from women who participated in the previous qualitative study (Lasater et al., 2018). After obtaining verbal informed consent, the adapted screening instrument (description below) was orally administered in Bambara by two trained female Malian research assistants with university degrees.

### 2.3. Instrument selection and adaptation

The study and identification of the local perinatal depression syndrome, *dusukasi* is described in-depth elsewhere (Lasater et al., 2018). Based on our previous formative qualitative findings (Lasater et al., 2018), we selected two instruments to adapt: the EPDS (Cox et al., 1987) and the Hopkins Symptom Checklist (HSCL-25) (Derogatis, 1974). Adaptation of the screeners included translation using terminology from the qualitative data that best reflected the items in the screeners (Lasater et al., 2018). For example, the item ‘feeling low in energy, slowed down’ translates to ‘body is dead’, and ‘I have felt scared or panicky for no very good reason’ translated to ‘your mind will not sit’ in Bambara. Items included in the screeners that were not reflected in the qualitative data were translated into Bambara and independently back translated into English. Locally relevant concepts identified in the formative study and not already represented on these screeners, such as ‘feeling your heart is broken or pained’ were added (Lasater et al., 2018). In many instances items on the EPDS and HSCL reflected the same concept but with differences in phrasing. Through consultation with our Malian research assistants and a local linguist, we retained the item/phrasing that would be best understood among Malian women. The preliminary 34-item instrument included 25 items from the HSCL, three items from the EPDS and six local items (Table 1). Rather than adapting the EPDS response scale options which varied by item and was thought would be difficult for women to understand, we chose to adapt the response options from the HSCL and make minor wording revisions in an effort to make the response options as consistent and simplistic as possible. The instrument was pretested among 42 women in four villages, resulting in slight wording modifications and the removal of five items. The final adapted instrument therefore consisted of 29 items. For each item, respondents were asked, “Over the past two weeks, how often have you felt _______?” using 4 response options (always; often; sometimes; never). We calculated a symptom score for each respondent by averaging item responses.

To assess functional impairment, we also included 7 items assessing difficulty completing typical daily activities (Table 2). These items were selected based on the findings of the earlier qualitative phase of the study where we asked women to list all of the activities women with young children frequently do to care for their family, themselves, and community (Applied Mental Health Research Group, 2013; Bolton & Tang, 2002). For each item, respondents were asked, “Over the past two weeks, how much difficulty have you had _______?“, using a five-point Likert scale (no difficulty; very little difficulty; a moderate amount of difficulty; a lot of difficulty; so much difficulty that I can’t do it) and a “not applicable” option. We also developed pictorial cues to accompany the functionality response options. These images show a woman carrying increasingly larger bundles of firewood on her head, with the first image showing a woman balancing wood on her head with no difficulty and last image showing a woman with such a large bundle of wood that she can’t stand up. We calculated a functional impairment score for each respondent by averaging responses using simple mean imputation to account for item level missingness. If a respondent indicated “not applicable” to 40% or more of the functional impairment items, we treated their score as missing.

### 2.4. Analysis

Exploratory factor analysis (EFA) was completed in mPlus 7.0 (Muthén LKaM, 1998–2012), and all other analyses were completed in Stata 14 (StataCorp, 2015).

#### 2.4.1. Dimensionality and scale revision

We conducted a principal components analysis (PCA) to assess dimensionality of the 29-item *dusukasi* instrument. Selection of the number of factors to include in an EFA was guided by the number of eigenvalues over one produced by the PCA, results of a parallel analysis, and the proportion of the variance explained by each component. For the EFA, we used weighted least squares estimation and a polychoric correlation structure. Considerations for item retention included loading predominantly on one factor with a magnitude >0.4, low uniqueness, and item-rest correlations.

#### 2.4.2. Reliability and internal consistency

We calculated Cronbach’s alpha to assess internal consistency among the items in the symptom scale revised through EFA. We considered a Cronbach’s alpha of greater than 0.7 and 0.8 to indicate adequate and good reliability, respectively (Nunnally & Bernstein, 1978).

#### 2.4.3. Construct validity

Given the scarcity of mental health providers in Mali and important differences between *dusukasi*, and a DSM diagnosis of depression, we felt the typical gold standard structured psychiatric interview was not an appropriate criterion (Bolton, 2001). Rather, we planned to use concordant reports between an individual and key informant (CHW) as the standard for comparison (Applied Mental Health Research Group, 2013; Bolton, 2001). After administering the full screening instrument, interviewers used a standardized script (Supplemental file 1) to ask women if they felt they experienced *dusukasi*. Interviewers probed as necessary to clarify the distinction of *small dusukasi*, problems or stress a woman may have from time to time, from *big dusukasi*, intense problems that are difficult to overcome. CHWs (n = 14) were blind to women’s scale responses and self-identification. We identified very low concordance between women’s and CHWs’ identifications (Kappa 0.03) in preliminary analyses, and therefore only used women’s self-identification to create known groups for assessing construct validity. Specifically, we compared mean symptom and functional impairment scores for women identified as likely experiencing based on their self-identification vs. those who self-identified as not having *dusukasi*, using a non-parametric Mann-Whitney given non-normal distribution of scale scores. Given the practical need for a cut-off score in clinical settings and identification of lower optimum cut off scores for perinatal depression screening in LMIC than those recommended for populations in HIC (Adewuya et al., 2006; Ali et al., 2016; Shrestha et al., 2016), we used an ROC curve to calculate sensitivity and specificity at various symptom scores using self-identification of *dusukasi* as an approximate criterion. We also assessed the relationship between functional impairment and symptom score.

#### 2.4.4. Creation of an abbreviated screening instrument

We conducted a graded response model item response theory (IRT) analysis to develop an abbreviated version of the scale. Consistent with previous work measuring depression in LMICs (Haroz et al., 2016a), we estimated the discrimination for each item and difficulty associated with each response category per item. We generated and examined item information functions and item characteristic curves to visually assess these parameters. We then used multivariate regression with the symptom items as predictors of functioning score to the assess local relevance of each item. We based our choice of items to retain on degree of discrimination (items that exceeded >1.35, a cutoff for high discrimination (Baker, 2001)), level of difficulty (items with varied locations on the latent trait at which probability of endorsement is 50%), and prioritization of items with a high degree of local relevance. Additionally, we considered respondent feedback from the piloting of the measure and content validity. Having selected a subset of items, we repeated the validity and internal consistency analyses using the newly shortened scale. Additionally, we generated test information functions curves for both versions of the scale to examine relative precision and test characteristic curves to assess expected *dusukasi* scale score at different points along the latent trait. We also generated a scatterplot to compare observed and expected scores.

## 3. Results

We pretested the preliminary screen among 42 women in four villages, and then administered the adapted *dusukasi* screener to 180 women in 13 villages (separate women and villages from pretesting). Approximately one third (n = 55, 31%) of the women were pregnant at the time of interview.

### 3.1. Pretesting

Pretesting of the instrument resulted in slight wording modifications and the removal of five items. Women described feeling uncomfortable when asked the item assessing sexual interest and said they did not like being asked this question. We proceeded to drop this item. Women also had a very hard time understanding two items, “no longer interested in anything”, and “feeling joyful when thinking about the future”. Both of these items required extensive explanations among almost all participants which ultimately still may not have been understood and women frequently expressed that they “don’t think like that” regarding looking to the future with joy. Women also identified repetitive items including two items asking about feelings of loneliness and feeling isolated or distant from others, and two items asking about feeling trapped and feeling like things are getting on top of you. In these instances we retained the items “feeling lonely” and “things have been getting on top of me” and dropped the items “feeling isolated or distant from others” and “feeling trapped”. Women also had a hard time understanding the visual cues that accompanied the functional impairment scale describing the visuals as confusing and distracting. We therefore proceeded to drop the visual cues.

### 3.2. EFA

PCA produced seven eigenvalues greater than one (11.10, 1.82, 1.71, 1.28, 1.19, 1.16, 1.05) that explained 67% of the variance. The first eigenvalue explained 38% of the variance. The results of the parallel analysis supported these findings favoring a single factor model. In a one-factor EFA, all but one item (“I have been able to laugh and see the funny side of things”) appeared to load above 0.4 onto the single factor (Table 1). As a sensitivity analysis, we ran a two-factor EFA (Supplemental file 2). Inclusion of a second factor resulted in multiple items loading poorly or splitting between the factors, suggesting that we were unnecessarily parsing a single underlying factor. We therefore proceeded to treat the screening instrument as a single 28-item scale (i.e., without the item “I have been able to laugh”) measuring one underlying construct of distress.

### 3.3. Scale shortening

Results from the IRT analysis of the 28 *dusukasi* items are presented in Table 3 with items retained for the shortened screener in bold. Overall, item discrimination was high (mean discrimination = 1.40, standard deviation 0.32), with no low performing items and 17 (61%) items having a high to very high discrimination. Non-never endorsement of having “headaches” was the item with the lowest difficulty parameter (−1.78), and suicidal ideation had the highest estimated difficulty parameter of any item (range from 2.75 to 5.27). All items retained for the shortened screener had high discrimination (>1.35), except for thoughts of ending your life; feelings of loneliness; and crying easily. We retained the item measuring suicidality because of its clinical relevance (in ascertaining safety) and relatively high difficulty, as we felt it was important for the scale to be able to distinguish among women experiencing higher levels of *dusukasi* in order to be sensitive to change over time. We retained loneliness and crying easily because of their frequent mention in the qualitative phase (Lasater et al., 2018). We dropped several items with high discrimination (feeling tense, feeling panicked, and feeling of being good for nothing) because of a lack of salience in the qualitative phase and redundancy in difficulty parameters.

### 3.4. Internal consistency

Cronbach’s alpha for the 22 items taken from the HSCL-25 was 0.89, while the alpha for the five locally generated items was 0.78. We did not assess internal reliability for the EPDS items separately, as only three were retained in the final scale. The alpha for the 7-item functional impairment scale was 0.86. Cronbach’s alpha for the full 28-item depression and anxiety symptom scale was 0.92 and 0.89 for the shortened 16-item scale.

### 3.5. Construct validity

Mean symptom score was 0.57 points greater among women who self-identified as having *dusukasi* (n = 87) than those who did not (n = 93) (Table 4). Using Mann-Whitney, this difference was found to be significant (p < 0.001). ROC curve analysis approximated a symptom score of 1.02 as an optimal cut-off point for determining caseness balancing sensitivity (0.68) and specificity (0.78). The difference in the mean score on the shortened symptom scale between women who identified as cases and non-cases was slightly greater than the full-scale score (0.64, p < 0.001). The optimal cut-off point identified using the shortened scale was approximately equivalent to the full scale (1.02), with a slightly higher sensitivity (0.71) and lower specificity (0.75).

When women were categorized by their reported level of functional impairment, mean symptom score increased in a dose-response manner with level of functional impairment (Table 4). Results from a global test produced by an ANOVA of symptom score by functional impairment category indicated that the symptom score among at least one group of women was different from another (F = 34.07, p < 0.001). Functional impairment and symptom scores also exhibited a strong positive correlation (Spearman’s rho = 0.62). The shortened symptom scale’s relationship to functional impairment was of similar strength and direction as the full scale (Spearman’s rho = 0.61).

## 4. Discussion

We demonstrated evidence of construct validity for a brief screening instrument for a locally described perinatal mental health syndrome (*dusukasi*) and a measure of functional impairment among perinatal women in Mali. EFA revealed a single underlying factor structure for *dusukasi*, encompassing established symptoms of depression and anxiety and local presentations of distress. Our functional impairment scale demonstrated good internal consistency and correlated positively and strongly with *dusukasi* symptom score. Using IRT, we substantially shortened the adapted screening instrument from 28 to 16-items without diminishing the scale’s internal consistency, ability to distinguish between women who self-identified as having or not having *dusukasi*, or association with functional impairment.

After piloting, we only retained three EPDS items on our instrument, one of which was later dropped due to a weak factor loading. Similar to findings from validation studies in other LMICs, items about sexual interest (Haroz et al., 2016a), looking forward to the future, and being able to see “the funny side of things” were problematic (Hanlon et al., 2008; Tesfaye et al., 2010). Our findings are also concordant with a recent systematic review of validation studies of the EPDS in LMICs, which found generally questionable results related to sensitivity and specificity, and positive predictive values of less than 80% (Shrestha et al., 2016). Though not specifically designed to assess perinatal women, we retained many items from the HSCL depression and anxiety sub-scales, as the wording was easily adapted and understood with our study population. While the HSCL sub-scales had a good fit with our qualitative data, they did require local adaptation and the addition of five local items to the adapted screener based on our prior qualitative data, which exhibited higher discrimination in the IRT analysis. In the Democratic Republic of Congo, Bass et al. (Bass et al., 2008) found that their locally adapted instrument for postpartum depression achieved greater reliability than either the EPDS or HSCL in their original forms. As such, in other low resource setting the HSCL may be a good starting point for adapting and validating local measures as has been demonstrated in the literature (Bass et al., 2008; Haroz et al., 2016a), but future research examining the HSCL and its adaptation is needed.

Our results suggest substantial overlap among symptoms of perinatal depression and anxiety which was also reflected in our previous qualitative study (Lasater et al., 2018). A recent systematic review of the qualitative literature on the experience of depression demonstrates a high comorbidity between symptoms of these two disorders cross culturally (Abas & Broadhead, 1997; Bener et al., 2012; Das-Munshi et al., 2008; Haroz et al., 2016b; Kaaya et al., 2002). This is consistent with the diagnosis of postpartum depression in HIC where clinicians have frequently observed women experiencing symptoms of anxiety when making depression diagnoses (Kumar & Robson, 1984; Wenzel et al., 2001). Such findings underscore that a focus on depression alone by healthcare providers, rather than CPMDs more broadly, could result in under-identification of perinatal women in need of supportive services.

Given the progress that has been made towards ensuring women in LMIC attend antenatal care and have a skilled birth attendant at their birth, the perinatal period represents an opportunity for healthcare workers to identify women and infants at risk of poor health outcomes due to CPMDs (Patel et al., 2009). Our findings on the lack of concordance between women’s self-identification as having *dusukasi* and community health worker’s identification demonstrates the challenge of asking health workers to identify women without giving them appropriate tools to do so. Multiple challenges, including shortages of providers, impede widespread implementation of screening for CPMDs by providers in primary and maternal healthcare settings even when tools are available (Hanlon et al., 2014; Larsen et al., 2021; Rochat et al., 2013; van Heyningen et al., 2019). Therefore, in rural areas where health facilities are limited and rushed providers may face high patient volumes, there is an urgency for screening tools that are short, sensitive, and user-friendly among providers with a range of educational levels (Chorwe-Sungani & Chipps, 2017; Larsen et al., 2021; Rochat et al., 2013). In South Africa, Rochat and colleagues found that a 5-item version of the EPDS exhibited the best overall psychometric performance as evaluated by ROC curves and Cronbach’s reliability measures, and improved specificity than the longer 10- or 7-item versions (Rochat et al., 2013). While our scale is 16 items, we substantially shortened a scale that incorporated depression, anxiety, and local symptoms, without losing accuracy. Given the range of difficulty of items on our scale, we hypothesize that it will also be feasible for use to detect change over time by maternal healthcare providers in Mali.

The process we described for developing and adapting a perinatal depression measure specific to the Malian context does not require an overabundance of resources but can help produce a more sensitive and specific measurement. Even in instances when local conceptions of mental health overlap or are in some ways similar to measures developed in HIC, there may still be key concepts, symptoms or signs of common mental health problems experienced locally that should be included not only improve measurement but also relevance to perinatal women in a specific context. For example, three key local items that we developed (mind is wandering or distracted; heart is broken or pained; and talking to yourself) could conceivably be understood to mean the same as items assessing distraction, sadness, or feeling like you have no one to talk to (Lasater et al., 2018). However, our qualitative study provided a contextualized understanding of these local items that allowed us to see just how distinct they were from description of similar ideas from HIC. Not surprisingly, these local items produced some of the highest factor loadings (0.75, 0.72, 0.71, respectively) in our analyses, highlighting the utility of this process. The Design Implementation Monitoring and Evaluation (DIME) manual, from which our methods were derived, elaborates on our processes in great detail, providing a step-by-step road map for other researchers to develop valid and reliable measures, firmly rooted in specific cultural context (Applied Mental Health Research Group, 2013).

Several limitations should be considered when interpreting the findings of this work. Unlike other efforts to adapt screeners for LMICs, information on local language and context collected during early qualitative research in Mali heavily informed the adaption of our screening instrument. While this process was critical for developing a tool to accurately identify women experiencing CPMDs (Bass et al., 2007), it likely limits the generalizability of these scales to other LMIC, particularly urban populations where daily living may be substantially different. Future research should explore the extent to which urbanicity effects the psychometric properties and validity of these tools.

Given the cross-sectional nature of this study, we are unable to establish temporality of symptoms of dusukasi. While 33% of our participants were pregnant at the time of the study, we were unable to assess symptom onset, whether before pregnancy, during pregnancy, in the immediate first 6-weeks postpartum, or later. In light of this limitation, our measure for dusukasi may not be entirely specific to the perinatal period, however, it is highly relevant to the experience of depression in the perinatal period. Future research should longitudinally examine women’s mental health trajectories beginning at start of antenatal care through two years postpartum. Moreover, given the high comorbidity of anxiety and depression across populations, future research should examine the validity of this measure among women outside of the perinatal period.

Another important limitation of this study is the lack of a ‘gold standard’ when assessing validity. Unlike other validation studies performed in LMIC (Baggaley et al., 2007; Chibanda et al., 2010; Hanlon et al., 2008), it was not feasible to use professional diagnosis or structured diagnostic interview as a criterion. This is because diagnoses by these methods would not match local definitions of *dusukasi* described in our qualitative work and the few Malian mental health specialists available to conduct structured diagnostic interviews. While we strived to define caseness by the concordance of women’s self-assessment with that of their CHWs, concordance was low, likely due to the nature of CHWs contact with the women, stigma surrounding symptoms, and a lack of training on mental health conditions. While our findings support the construct validity of the scale, ROC analyses presented here are exploratory given limitations in case identification in this context. In the future, additional studies of the validity of our instrument should be conducted using a different key informant, such as a sister, cousin, or friend who may be more familiar with her psychosocial health. However, this challenge also highlights the importance of developing a useable tool for CHWs and providers to identify CPMDs, rather than relying solely on unstructured observations and opinions in determining who needs care. In Mali and other resource constrained settings, there will likely also be a need for standardized, competency-based training for providers and CHWs to permit screening and detection of women who may need more specialized support (Ali et al., 2016; Honikman et al., 2012; Rochat et al., 2013; van Heyningen et al., 2019). It is possible that low concordance between CHWs and women was due to a woman’s lack of insight in her condition or unwillingness to report, our construct validity analyses suggest that women who report more symptoms and functional impairment were also more likely to identify themselves as experiencing dusukasi. However, it is important to note that as all measures and caseness were self-reported by the woman, our construct validity analyses may be subject to common methods bias (Podsakoff et al., 2003), in which women who report symptoms of dusukasi may also be more likely to self-report themselves as experiencing Dusukasi, biasing the observed association away from the null.

## 5. Conclusions

In summary, we developed a locally informed screening tool for *dusukasi*, a local perinatal depression and anxiety syndrome in rural Mali and found support for its construct validity. Together with our development of a functional impairment scale, these measures can help ensure that women who are most impacted by CPMDs can be identified and prioritized for services in a resource limited setting. Our findings and the process we describe underscores the utility in understanding local conceptions of mental health for more sensitive and locally relevant measurement of mental health.

## Supplementary Material

1

2

## Figures and Tables

**Table 1:** Exploratory Factor Analysis results (n = 180).

Item	Item Source	Factor Loading	Uniqueness
Suddenly scared for no reason	HSCL-A	0.66	0.41
Feeling fearful	HSCL-A	0.55	0.65
Faintness, dizziness, or weakness	HSCL-A	0.49	0.74
Nervousness or shakiness inside	HSCL-A	0.56	0.57
Heart pounding or racing	HSCL-A	0.52	0.68
Trembling	HSCL-A	0.56	0.68
Feeling tense or keyed up	HSCL-A	0.62	0.50
Headaches	HSCL-A	0.52	0.73
I have felt scared or panicky for no very good reason	HSCL-A^a^; EPDS	0.61	0.62
Feeling restless, can’t sit still	HSCL-A	0.67	0.55
Feeling low in energy, slowed down	HSCL-D	0.73	0.47
Blaming yourself for things	HSCL-D	0.41	0.83
Crying easily	HSCL-D^a^; EPDS	0.61	0.62
Poor appetite	HSCL-D	0.67	0.55
Difficulty falling asleep, staying asleep	HSCL-D^a^; EPDS	0.61	0.63
Feeling hopeless about the future	HSCL-D	0.66	0.57
Feeling sad	HSCL-D^a^; EPDS	0.64	0.58
Feeling lonely	HSCL-D	0.56	0.69
Worrying too much about things	HSCL-D^a^; EPDS	0.74	0.42
Feeling everything is an effort	HSCL-D	0.68	0.36
Feelings of worthlessness	HSCL-D	0.64	0.54
I have been able to laugh and see the funny side of things	EPDS	0.21	0.86
Things have been getting on top of me	EPDS	0.50	0.72
Talking to yourself	Local	0.71	0.49
Finding it difficult to talk to others	Local	0.64	0.57
Feeling your heart is broken or pained	Local	0.72	0.48
Your mind is wandering or distracted	Local	0.75	0.42
Becoming angry easily	Local	0.65	0.53
Thoughts of ending your life	HSCL-D	0.43	0.76

a Similar wording on HSCL and EPDS but retained HSCL wording.

**Table 2:** Functional impairment items included in the adapted perinatal depression screening instrument.

1. Cooking
2. Cleaning the house
3. Washing clothes
4. Taking care of the children
5. Collecting firewood
6. Bathing
7. Working in the fields (agriculture)

**Table 3:** Item Response Theory estimation of item discrimination and difficulty (n = 180).

Item	Discrimination	Difficulty^a^		
	a	*b*_1_	*b*_2_	*b*_3_
You felt afraid for no reason (A1)^b^	1.41 (0.21)	−0.84 (0.18)	0.79 (0.17)	1.62 (0.25)
You felt scared (A2)	1.15 (0.21)	0.02 (0.16)	1.72 (0.31)	3.78 (0.72)
You had a feeling of dizziness or weakness (A3)	0.96 (0.17)	−1.20 (0.27)	0.90 (0.23)	2.25 (0.42)
You feel nervous (A4)	1.08 (0.19)	−1.50 (0.28)	0.70 (0.19)	2.19 (0.37)
You felt that your heart beat abnormally fast (A5)	1.04 (0.19)	−0.24 (0.18)	1.08 (0.23)	2.37 (0.43)
You had the feeling of trembling (A6)	1.16 (0.24)	1.04 (0.22)	2.06 (0.39)	3.51 (0.72)
You felt tense (A7)	1.36 (0.21)	−0.72 (0.18)	0.96 (0.18)	2.09 (0.31)
You had headaches (A8)	1.03 (0.18)	−1.78 (0.32)	0.15 (0.17)	1.07 (0.23)
You felt panicked (A9)	1.42 (0.28)	1.07 (0.20)	1.83 (0.30)	2.79 (0.50)
You felt agitated (A10)^b^	1.62 (0.26)	0.21 (0.13)	1.13 (0.18)	1.99 (0.28)
You lack energy (A11)^b^	1.76 (0.24)	−1.42 (0.20)	0.27 (0.13)	1.29 (0.18)
You felt a sense of guilt (A12)	0.75 (0.17)	−0.22 (0.23)	2.21 (0.51)	4.07 (0.94)
You were crying easily (A13)^b^	1.34 (0.22)	−0.004 (0.15)	1.27 (0.15)	2.20 (0.34)
You had lost your appetite (A14)^b^	1.51 (0.22)	−0.94 (0.18)	0.33 (0.14)	1.57 (0.23)
Your sleep was disturbed (A15)^b^	1.37 (0.21)	−0.37 (0.16)	0.67 (0.16)	1.75 (0.27)
You felt desperate (A16)^b^	1.53 (0.25)	0.18 (0.14)	1.97 (0.29)	2.40 (0.36)
You felt depressed (A17)^b^	1.48 (0.22)	−0.80 (0.17)	0.88 (0.17)	2.54 (0.37)
You had the feeling of loneliness (A18)^b^	1.15 (0.21)	0.02 (0.16)	1.16 (0.23)	2.26 (0.38)
You worry too much (A19)^b^	1.86 (0.26)	−0.67 (0.15)	0.18 (0.12)	0.72 (0.14)
Everything was an effort for you (A20)	1.63 (0.23)	−0.46 (0.15)	0.66 (0.14)	1.78 (0.24)
You had the feeling of being good for nothing (A21)	1.50 (0.24)	−0.14 (0.14)	1.05 (0.19)	1.88 (0.28)
You tended to feel overwhelmed by things (A23)	1.02 (0.19)	−0.26 (0.19)	1.53 (0.29)	2.77 (0.50)
You were talking to yourself (A24)^b^	1.74 (0.26)	−0.08 (0.13)	0.80 (0.13)	1.79 (0.24)
It was difficult for you to talk to others (A25)^b^	1.49 (0.22)	−0.43 (0.15)	0.76 (0.16)	2.30 (0.33)
You felt your heart was broken or pained (A26)^b^	1.87 (0.25)	−1.06 (0.17)	0.49 (0.13)	1.74 (0.22)
Your mind wanders or is distracted (A27)^b^	2.13 (0.30)	−0.05 (0.12)	0.82 (0.14)	1.69 (0.21)
You get angry easily (A28)^b^	1.51 (0.22)	−1.02 (0.19)	0.87 (0.17)	1.97 (0.28)
You had thoughts about ending your life (A29)^b^	1.09 (0.39)	2.75 (0.78)	4.23 (1.33)	5.27 (1.83)

a b^1^ corresponds to comparing a response of “0” to a response of “1”, “2”, or “3”; b^2^ corresponds to comparing a response of “0” or “1” to a response of “2” or “3”; b^3^ corresponds to comparing a response of “0”, “1”, or “2” to a response of “3”

b Bolded items indicate items included in the shortened scale.

**Table 4:** Construct validity and internal consistency of full and abbreviated *dusukasi* scale (n = 180).

	Full Symptom Scale	Shortened Symptom Scale
Symptom score		
Cases, Mean (SD)	1.26 (0.48)	1.32 (0.06)
Non-Cases, Mean (SD)	0.69 (0.43)	0.69 (0.05)
Difference, Mean (SD)	0.57 (0.07)^a^	0.64 (0.07)^a^
Odds of caseness associated with a one unit increase in symptom score	14.26 (6.05)^a^	12.57 (5.01)^a^
Mean Score (SD) by Functional Impairment Quartile		
Bottom	0.47 (0.30)	0.44 (0.32)
Lower-Middle	0.86 (0.44)	0.89 (0.52)
Upper-Middle	1.24 (0.49)	1.27 (0.56)
Top	1.31 (0.44)	1.37 (0.45)
Correlation with Functioning Score	0.62^a^	0.61^a^
Area under the curve (95% CI)	0.81 (0.75, 0.87)	0.81 (0.75, 0.87)
Optimal Cut Point	1.02	1.03
Sensitivity at cut point	0.68	0.71
Specificity at cut point	0.78	0.75
Cronbach’s alpha	0.92	0.89

a p < 0.001.

## Data Availability

The dataset analyzed by the current study is available from the corresponding author on reasonable request.
