# Supplementary material for: Reliability and validity of a perinatal depression screening instrument in rural Mali
Source: SSM Ment Health. Author manuscript; Available in PMC 2023 Jan 12. (PMC9835090; doi:10.1016/j.ssmmh.2021.100059)
Supplement: 1 [file NIHMS1861029-supplement-1.docx]

**Supplemental file 1.** Standardized script for *dusukasi* self-report

*This questionnaire focused on the different problems that women who are pregnant or who have children under the age of two may have. We have conducted other studies here in Sélingué, where women have mentioned "dusukasi" as a problem that women here may have. I want to ask you if you think you have dusukasi. The dusukasi that I am talking about is the intense dusukasi - you sleep with it, you wake up with it - it is always there, and you can't find a solution. Do you think you have this "dusukasi"?*

*- Additional Probe/Explanation: Sometimes we talk about "dusukasi" as something small or temporary that we can have. Everyone has problems, everyone has this type of "small dusukasi" from time to time. The big dusukasi I'm talking about is deeper and much harder to overcome.*

Answers were recorded as “yes” or “no”.
