# Supplementary material for: Reliability and validity of a perinatal depression screening instrument in rural Mali
Source: SSM Ment Health. Author manuscript; Available in PMC 2023 Jan 12. (PMC9835090; doi:10.1016/j.ssmmh.2021.100059)
Supplement: 2 [file NIHMS1861029-supplement-2.docx]

| **Supplemental File 2:** Exploratory Factor Analysis Results (n=180) | | | | | |
| --- | --- | --- | --- | --- | --- |
|  | *Item Source* | | | *Factor Loadings* | |
| *Item* | |  | | *Factor 1* | *Factor 2* |
| Suddenly scared for no reason | | | HSCL-A | 0.76 | 0.01 |
| Feeling fearful | | | HSCL-A | 0.49 | 0.13 |
| Faintness, dizziness, or weakness | | | HSCL-A | 0.36 | 0.19 |
| Nervousness or shakiness inside | | | HSCL-A | 0.67 | -0.01 |
| Heart pounding or racing | | | HSCL-A | 0.50 | 0.09 |
| Trembling | | | HSCL-A | 0.09 | 0.50 |
| Feeling tense or keyed up | | | HSCL-A | 0.67 | 0.05 |
| Headaches | | | HSCL-A | 0.11 | 0.44 |
| I have felt scared or panicky for no very good reason | | | HSCL-A*; EPDS | 0.35 | 0.32 |
| Feeling restless, can’t sit still | | | HSCL-A | 0.24 | 0.49 |
| Feeling low in energy, slowed down | | | HSCL-D | 0.18 | 0.59 |
| Blaming yourself for things | | | HSCL-D | 0.06 | 0.37 |
| Crying easily | | | HSCL-D*; EPDS | 0.35 | 0.33 |
| Poor appetite | | | HSCL-D | 0.17 | 0.55 |
| Difficulty falling asleep, staying asleep | | | HSCL-D*; EPDS | 0.11 | 0.53 |
| Feeling hopeless about the future | | | HSCL-D | 0.20 | 0.51 |
| Feeling sad | | | HSCL-D*; EPDS | 0.00 | 0.65 |
| Feeling lonely | | | HSCL-D | 0.16 | 0.44 |
| Worrying too much about things | | | HSCL-D*; EPDS | -0.01 | 0.77 |
| Feeling everything is an effort | | | HSCL-D | -0.35 | 1.00 |
| Feelings of worthlessness | | | HSCL-D | -0.10 | 0.75 |
| I have been able to laugh and see the funny side of things | | | EPDS | -0.34 | 0.52 |
| Things have been getting on top of me | | | EPDS | -0.06 | 0.57 |
| Talking to yourself | | | Local | 0.34 | 0.43 |
| Finding it difficult to talk to others | | | Local | 0.03 | 0.64 |
| Feeling your heart is broken or pained | | | Local | 0.17 | 0.60 |
| Your mind is wandering or distracted | | | Local | 0.09 | 0.70 |
| Becoming angry easily | | | Local | 0.55 | 0.18 |
| Thoughts of ending your life | | | HSCL-D | 0.51 | -0.03 |

^* Similar wording on HSCL and EPDS but retained HSCL wording^
